# Unqualified Practice and the Law

**Published:** 1896-06-13

**Authors:** 


### Unqualified Practice and the Law.

The letters of Mr. Victor Horsley and others, which have recently appeared in the *Times*, on the subject of Regina v. Priestley, demonstrate once more with convincing certainty the weakness of the medical profession as a political power. We are convinced that that weakness is an injury to the nation. Students of history know well how constitutional rulers have always distrusted large separate organisations of educated men, bodies like the Church, for example; and with reason. Such bodies have often contained within themselves germs of political revolutions.

But we desire to point out with great earnestness that medicine does not seek political influence except for one especial object, and that a non-political object. When medicine, and in a very pronounced sense scientific medicine, sets itself upon influencing the Legislature, its sole object is the public health and well-being. These things are said because there seems to be an amount of jealous feeling displayed against legitimate medical influence in so many quarters that the public health and the public interests are seriously injured thereby.

Now in the matter of qualified as against unqualified practice we are brought face to face, by the decision in the case of Regina v. Priestley, with the fact that men and women possessing no knowledge whatever of medicine may, nevertheless, openly practise medicine as much as they please provided they do not assume medical titles. Nay, it would seem now that they may even assume medical titles if they take care to assume only such as are not registrable in this country. In short, the law which was designed to protect the public against the very serious danger of submitting its bodily ailments to persons who do not and cannot know anything either about the body or its ailments is for practical purposes a dead letter.

The questions that arise in the mind on seriously considering this state of things are something like these: Would unlimited medical quackery be a very great danger to the public? Is science better than ignorance? Is the hardly acquired knowledge of the modern medical man of real service to the community? Is the nation any healthier than it was? Is it freer from such foul diseases as plagues, small-pox, cholera, and the like? Is the world on the whole more wholesome in body and mind and less superstitious than it formerly was? And if all these questions must be answered in the affirmative, must it not also be admitted that scientific medicine has conquered all this knowledge and science for us? And should it not be further admitted that if these be the fruits of modern medical science, then that science is worthy of public confidence and approval?

The more thoughtful public, there are many grounds for believing, sees these things as we see them. The *Times* newspaper, we are persuaded, very largely utters the educated opinion of the times, and its trumpet gives no uncertain sound when dealing with the subject of medical quackery. The hour, indeed, seems to have struck for some new and more resolute action for the suppression of unqualified practice. Of late the only action taken has been that of querulous complaints to the public through the Press and of faultfinding with the judges and other interpreters of the law. But the subject is not one for the decision of judges or other legal persons. It must be carried beyond them, into the region of general political principles. Judges are but administrators of the law as it stands. It is necessary to push forward into the region of first principles and to make our representations to the constructors, not to the administrators of the law.

In a word, the law as it stands, which is merely aimed at preventing the use of medical titles, is a pure futility, even when sternly administered, so far as the protection of the public against unqualified practice is concerned. When it was made it was a well-meant law, and its object was to suppress quackery. Well, it has failed, and the failure has been complete. Clearly, then, new and more effective legislation must be tried if the public is to be protected from the dangers and impositions of unqualified practice.

